# Superoxide dismutase@zeolite Imidazolate Framework-8 Attenuates Noise-Induced Hearing Loss in Rats

**DOI:** 10.3389/fphar.2022.885113

**Published:** 2022-05-16

**Authors:** Yan Zhang, Qing Li, Chengzhou Han, Fang Geng, Sen Zhang, Yan Qu, Wenxue Tang

**Affiliations:** ^1^ Department of Otolaryngology, Hebei Medical University, Shijiazhuang, China; ^2^ Department of Otolaryngology, Tangshan People’s Hospital, Tangshan, China; ^3^ Department of Molecular Pathology, Application Center for Precision Medicine, The Second Affiliated Hospital of Zhengzhou University, Zhengzhou, China

**Keywords:** SOD@ZIF-8, biomimetic mineralization, reactive oxygen species, noise-induced hearing loss, inflammation

## Abstract

Reactive oxygen species (ROS) and inflammation have been considered major contributors to noise-induced hearing loss (NIHL) that constituted a public health threat worldwide. Nanoantioxidants, with high antioxidant activity and good stability, have been extensively used in the study of ROS-related diseases. In this study, we constructed a superoxide dismutase (SOD)@zeolite imidazolate framework-8 (ZIF-8) nanoparticle based on biomimetic mineralization and applied it to a rat model of NIHL. Our results showed that SOD@ZIF-8 effectively protected the animals from hearing loss and hair cell loss caused by noise. ROS, oxidative damage, and inflammation of noise-damaged cochlea were attenuated considerably after SOD@ZIF-8 administration. Importantly, we found that SOD@ZIF-8 achieved nanotherapy for NIHL in rats via a primary effect on the Sirtuin-3 (SIRT3)/superoxide dismutase2 (SOD2) signaling pathway without obvious adverse side effects. Therefore, our study is expected to open up a new field for NIHL treatment, and lay a foundation for the application of nanomaterials in other ROS-related inner ear diseases.

## 1 Introduction

Noise-induced hearing loss (NIHL) is a major problem for many patients worldwide, including many adolescents, and severely decreases the quality of life (Nelson et al., 2005; Basner et al., 2014; Alnuman and Ghnimat, 2019; Chen et al., 2020). As reported by the World Health Organization, the NIHL prevalence is ∼16% in the adult population worldwide (Nelson et al., 2005), and it imposes an enormous economic burden at a societal level, primarily attributed to the healthcare system burden and productivity losses. In the last 2 decades a growing number of experimental observations significantly increased our knowledge about noise induced multi-target cochlear damage, main molecular pathways involved in NIHL pathogenesis and effects of class I/II antioxidants on both cochlear and central auditory NIHL effects. Various antioxidants play protective roles in NIHL, but maybe owing to their limited efficacy and unclear mechanism, they cannot be applied efficiently clinically.

It is extensively accepted that noise can induce the generation of excess number of free reactive oxygen (ROS), including superoxide anions (O_2_^−^) and hydroxyl radicals (·OH) (Henderson et al., 2006; Böttger and Schacht, 2013). The overproduction of ROS causes oxidative damage to deoxyribonucleic acid, proteins, and lipids, which can lead to apoptosis and necrosis. Studies have shown that ROS can increase the concentration of free calcium ions in cochlear hair cells (Kurabi et al., 2017) that may induce cytoplasmic ROS accumulation, trigger an excessive release of glutamate, and may also activate ROS-independent apoptotic and necrotic cell death pathways (Orrenius et al., 2003). ROS can generate proinflammatory cytokines that can further damage the cochlea (Fuentes- Santamaría et al., 2017; Paciello et al., 2018; Fetoni et al., 2019). Additionally, an overview of research trends and genetic polymorphisms for NIHL also indicated that inflammation was one of the important underlying mechanism of this disease (Miao et al., 2019).

Mn-superoxide dismutase (Mn-SOD), a subtype of SOD that is exclusively expressed in the intracellular mitochondrial matrix, is one of the endogenous tools used by mitochondria to scavenge ROS. Previous research has shown that oxidative stress-mediated histone methylation on the Mn-SOD promoter suppressed the expression of Mn-SOD (Wang et al., 2019a). Therefore, supplementation of antioxidant enzymes to the inner ear is a potential therapeutic strategy for NIHL. However, direct use of antioxidant enzymes as a therapeutic option is limited because of its short half-life, poor permeability across the cellular membranes, temperature and pH sensitivity, and hard-mass production (Banks, 2008; Yao et al., 2018). Recently, nanomaterials with intrinsic enzyme-like activities have already become the focus of numerous studies in multiple fields owing to their high ROS scavenging ability and anti-inflammatory activity (Wang et al., 2019b). They have shown significant therapeutic potential in various ROS-associated diseases, including inflammatory bowel disease (Zhao et al., 2019), colitis (Zhao et al., 2018), Parkinson’s disease (Singh et al., 2017), cancer (Shen et al., 2020), ischemic stroke (Mu et al., 2019), and ear inflammation (Yao et al., 2018), while there are rare few reports about it in the field of inner ear disease.

**FIGURE 1:**
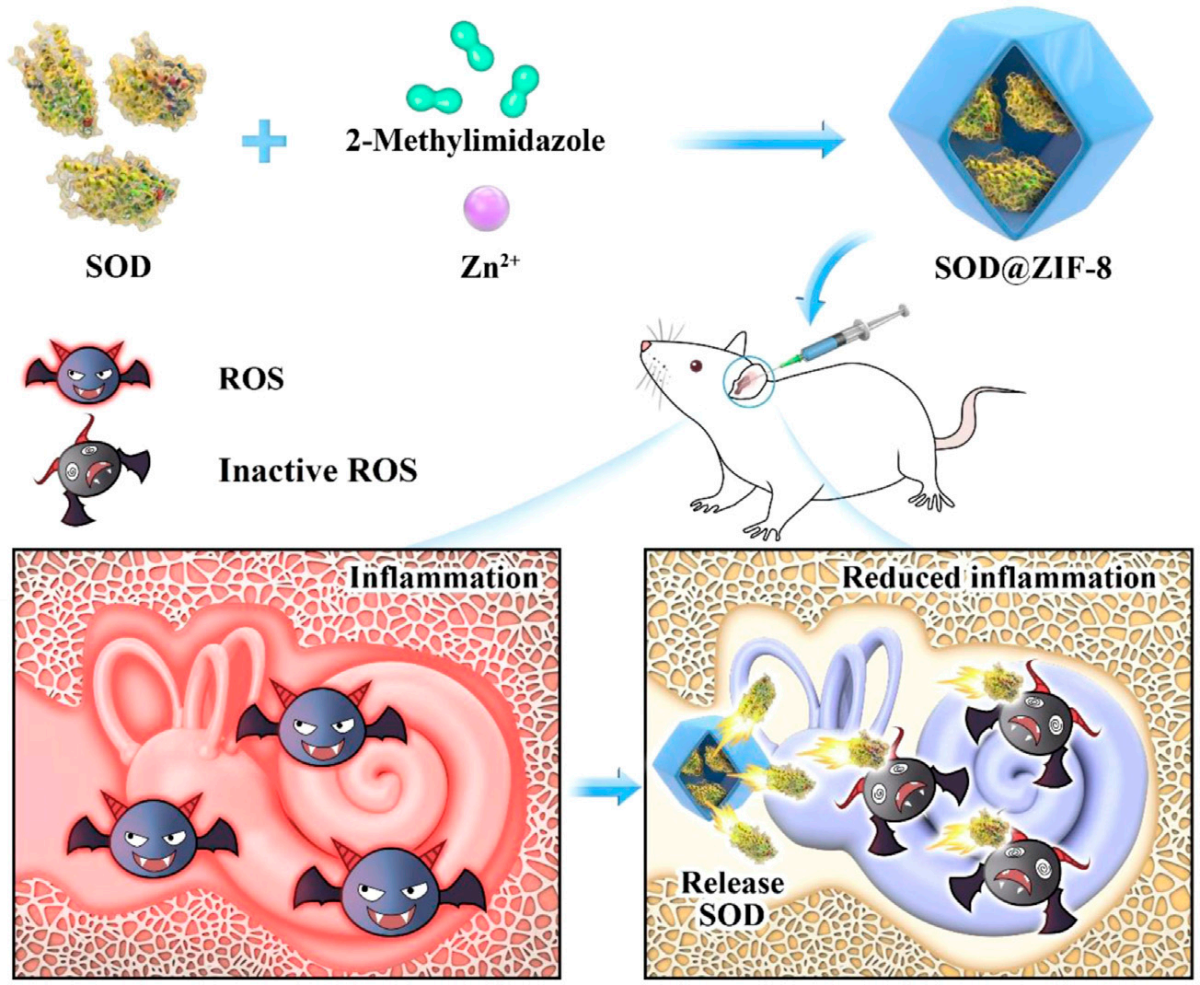
Schematic illustrating the synthetic process of superoxide dismutase (SOD)@zeolite imidazolate framework-8 (ZIF-8) and its use for the therapy of inner ear diseases associated with reactive oxygen species (ROS).

The clearance efficiency of ROS from most nanoantioxidants is limited owing to different synthetic materials contained by them and the techniques used to synthesize them. Metal-organic frameworks (MOFs) comprise unique porous materials characterized by high porosity, high-enzyme loading, large surface areas, and tunable functionality (Long et al., 2018; Ngwa, 2018). As a representative of MOFs, the porous nanomaterial zeolite imidazolate framework-8 (ZIF-8) has been recognized as an effective drug carrier owing to its nontoxic, biocompatible, excellent stability under physiological conditions or acidic environments (Zheng et al., 2016; Ito et al., 2019). In a previous study, we encapsulated an artificial enzyme mimic in ZIF-8 via a biomimetic mineralization approach (Jiang et al., 2017). This composite not only showed enhanced catalytic activity and favorable stability but also demonstrated easy separation characteristics of catalyst from the system and the ability to eliminate iron residues in the synthesized polymer. Based on the abovementioned information, we embedded Mn-SOD in ZIF-8 using the biomimetic mineralization approach and applied it for the first time in the treatment of NIHL. SOD@ZIF-8 was applied in the rat model of NIHL by locally injecting the animals with a single-dose administration in the round window niche (RWN). It exhibited good hearing protection from noise exposure by decreasing the auditory brainstem response (ABR) threshold value and reducing the damage of cochlear hair cells and connexin. Moreover, this nanomaterial reduced the level of cochlear ROS and lipid peroxidation, suppressed inflammation and apoptosis, and upregulated the Sirtuin-3 (SIRT3)/Mn-SOD signaling pathway. The experimental setting of this study was not primarily and extensively aimed to the documentation of SOD@ZIF-8 local and/or systemic side effects. The synthetic SOD@ZIF-8 will contribute to the treatment of ROS-associated inner ear diseases (Figure 1).

## 2 Materials and Methods

### 2.1 Materials and Preparation of SOD@ZIF-8

The SOD@ZIF-8 composites were constructed according to previous reports based on biomimetic mineralization. Briefly, 20 ml of 2-methylimidazole solution (45.68 mg/ml) was mixed with 20 ml of Zn(OAc)_2_ solution (8.76 mg/ml), 2 ml of methanol, and 10 ml of SOD solution (1 mg/ml). The compound was incubated at room temperature for 24 h, and solids were then collected by centrifugation and washed until no absorbance could be detected in the supernatant at 280 nm.

### 2.2 Animals and Round Window Membrane Administration

All research procedures were reviewed and approved by the Animal Experimental Ethical Committee at the Third Hospital of Hebei Medical University. Male adult Wistar rats (Beijing HFK, Beijing, China), weights: 200–250 g, ages: 2 months, with intact Preyer’s reflex, were used for this study. Seventy-two rats were randomly divided in two groups: 1) control animals (control group, *n* = 18), 2) noise-exposed animals and treated with 10 μl of SOD@ZIF-8 2 mg/ml suspension in the left ear (Noise- SOD@ZIF-8 group) and with vehicle (saline) in the right ear (noise–vehicle group) 1 day before noise exposure (*n* = 24). All rats were housed as three per cage in the experimental animal center of the Third Hospital of Hebei Medical University and were kept at temperatures in the range of 22–24°C and at relative humidity levels of 55 ± 5% on a 12 h light-dark cycle.

Rats were anesthetized by intraperitoneal injection of sodium pentobarbital (40 mg/kg), and the temperature was maintained at 37°C during surgery. We made a 1.5 cm retro-auricular incision and drilled a small hole in the bulla to expose the RWN under a microscope. We carefully applied 2 μl SOD@ZIF-8 (or vehicle) and applied it to the round window membrane (RWM) by using a microsyringe. The hole of bulla was then sealed with sterilized medical bone wax and the incision was closed with 4–0 mouse sutures.

### 2.3 Noise Exposure

One day after surgery, rats were exposed to broadband white noise at sound pressure levels (SPL) in the range of 120–125 dB via a custom-made sound chamber for 12 h/day for three consecutive days. The noise generation system consists of AW A61290M two-channel acoustic analyzer and 100W AWA5870B amplifier. Control rats were kept in silence within the same chamber for the same time. To avoid the effect of sound pressure by the adjacent animals, each rat was separated by a wire mesh. When in the sound chamber, rats were free to eat and drink.

### 2.4 Hearing Function Measurements

ABRs were recorded in a soundproof chamber before noise exposure and at 1, 3, 7, 14, and 3,028 days after exposure. Tone burst stimuli between 100 and 20 dB in 5 dB increments were generated, with a 0.2 ms rise/fall time and 1 ms flat segment at frequencies of 4, 8, 12, 16, 20, 24, 28, and 32 kHz, and the amplitude was specified by a sound generator and attenuation real-time processor and programmable attenuator (Tucker-Davis Technology, Alachua, FL, United States). We defined the lowest stimulus level (dB) that yielded a repeatable waveform-based onset as threshold values.

### 2.5 Immunostaining

Rats were decapitated after the administration of deep anesthesia, and the temporal bones were quickly removed on day 7 after noise exposure. Cochleae were fixed following their perfusion with 4% paraformaldehyde and were then further immobilized overnight at 4°C and decalcified in 10% ethylenediaminetetraacetic acid (EDTA) in 10 mM phosphate-buffered solution (PBS) at room temperature for 10–15 days. For surface preparation, cochlear sensory epithelia were microdissected from the cochleae and divided into apical, middle, and basal turn sections. For the frozen section, the cochleae were dehydrated with 10 and 30% gradient sucrose. Samples were then embedded in optimal cutting temperature compound and were then sectioned into 10 μm thick slices. The sections or cochlear sensory epithelia samples were washed in PBS and were then permeabilized in 0.3% Triton X-100, which contained 3% bovine serum albumin (BSA) in PBS at room temperature for 60 min. The samples were blocked in 10% goat serum in PBS at 37°C for 60 min and were incubated with a primary antibody (rabbit antimyosin-VIIa, 1:200, Proteus; rabbit anti-4-hydroxynonenal [4-HNE], 1:50, Abcam; rabbit anti-interleukin [IL]-1β, 1:100, GeneTex; rabbit anti-SIRT3, 1:50, ABclonal) at 4°C overnight. Samples were washed thrice in PBS and then incubated with Alexa-Fluor-labeled antirabbit secondary antibody (1:200) in the dark at room temperature for 90 min. After another wash in PBS, samples were counterstained with 4′6′-diamino-2-phenylindole (DAPI) (1:150) for 10 min in the dark. For dihydroethidium (DHE) staining, the cochlear specimens were incubated with 5 μM DHE (Beyotime) for 30 min at room temperature. Fluorescence images were acquired at two different magnification settings (20× or 40×) using a confocal laser scanning system (TCS SP5, Leica, Germany).

### 2.6 Western Blot Analysis

Immunoblotting procedures for Cx26, Cx30, 4-HNE, bax, bcl-2, interleukin (IL)-6, IL-1β, phosphonuclear factor kappa B (p-NF-κB), SIRT3, and SOD2 were performed, and four cochleae/groups were used in each procedure. Dissected cochleae (volute removed) were collected on ice. Total protein was extracted using a protein extraction kit (Invent, United States) and homogenized in ice-cold radioimmunoprecipitation assay buffer mixed with phosphatase inhibitor cocktails II and III and protease inhibitor (Yuan et al., 2015) and centrifuged at 12,000 revolutions per minute for 30 min at 4°C. Protein concentration was measured with the use of a bicinchoninic acid assay kit (Servicebio, China). Protein samples were separated by 8–12% sodium dodecyl sulfate-polyacrylamide gel electrophoresis and transferred to polyvinylidene fluoride membranes. Membranes were blocked in Tris Buffered Saline with Tween 20 (TBST) containing 5% dry milk (the membrane of p-NF-κB was blocked in TBST, which contained 5% BSA) for 2 h at room temperature, and were incubated overnight at 4°C with primary antibodies (anti-Cx26, Cx30, 1:1,000, Invitrogen; IL-6, IL-1β, SOD2, 1:1,000, GeneTex; p-NF-κB, bax, bcl-2, 1:500, Affinity; SIRT3, 1:1,000, ABclonal). Membranes were incubated with antirabbit fluorescent secondary antibody. The protein band pixel intensities were normalized by the actin band in each sample. The bands were visualized and analyzed using an Odyssey Infrared Imaging System (LICOR 9120; Li-COR, Lincoln, NE, United States).

### 2.7 Safety Testing of SOD@ZIF-8 in Rats

Before the above experimental rats were sacrificed, serum samples were collected for biochemical tests, including alanine aminotransferase (ALT), aspartate aminotransferase (AST), blood urea nitrogen (BUN), creatinine (CR), and creatine kinase (CK). Additionally, the main organs (including heart, brain, liver, spleen, and kidney) were harvested for pathological analysis following hematoxylin and eosin (H&E) staining.

### 2.8 Statistical Analysis

Results were presented as mean ± standard error of mean (SEM) or mean ± standard deviation. Data analysis and statistics were conducted using paired *t*-tests or Wilcoxon signed-rank tests and one-way analysis of variance.

## 3 Results and Discussion

### 3.1 Characterization of SOD@ZIF-8

**FIGURE 2:**
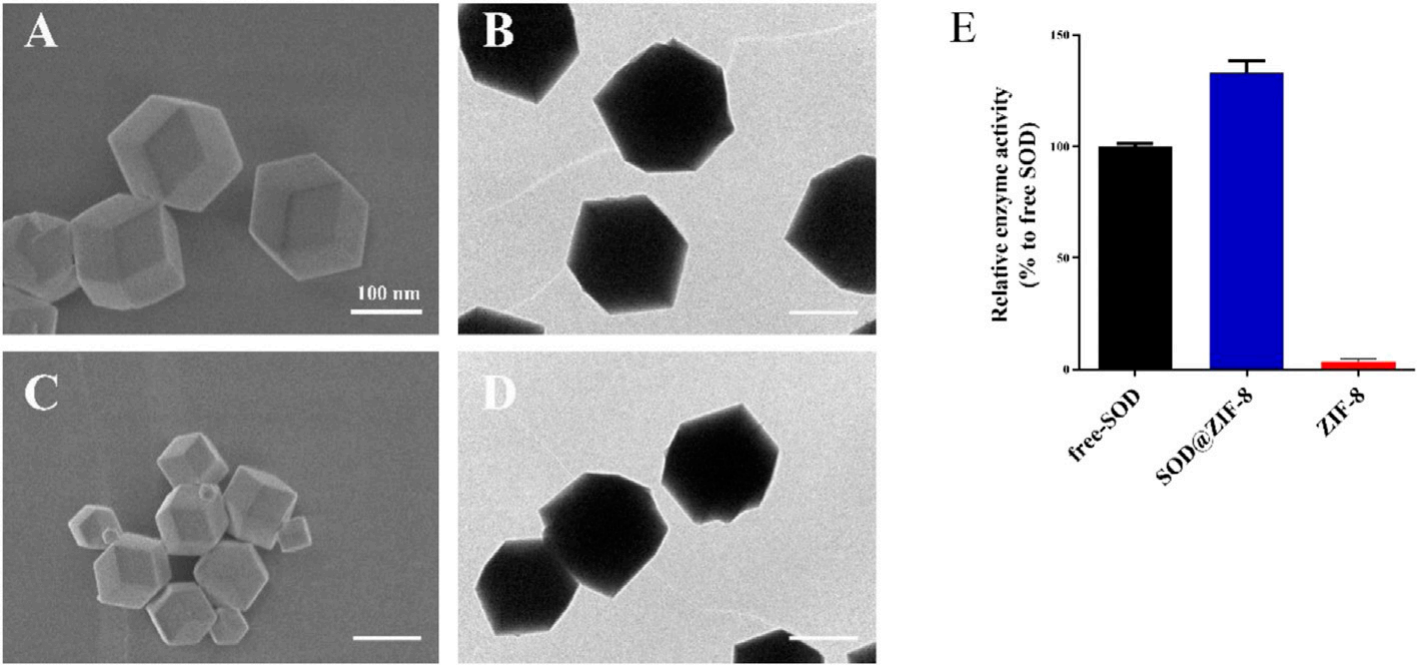
Scanning electron microscopy (SEM) **(A,C)** and transmission electron microscopy (TEM) **(B,D)** images of superoxide dismutase (SOD)@zeolite imidazolate framework-8 (ZIF-8) **(A,B)** and ZIF-8 **(C,D)**. Enzymatic activity quantified with the use of the nitroblue-tetrazolium-ultraviolet spectrum **(E)**.

The morphology of SOD@ZIF-8 assembling enzyme was characterized by scanning electron microscope (SEM) and transmission electron microscopy (TEM) (Figures 2A–D). SOD@ZIF-8 demonstrated a more uniform structure with regular dodecahedral structure, thus indicating that SOD could be responsible for the morphogenetic formation of the assembling enzyme owing to the coordination of SOD with ZIF-8. Furthermore, the enzymatic activity of SOD was determined based on the nitroblue tetrazolium assay (Figure 2E). Compared with free SOD, SOD@ZIF-8 assembling enzyme demonstrated an increasing activity that was probably attributed to the dispersion effect of ZIF-8.

### 3.2 SOD@ZIF-8 Attenuates Auditory Threshold Shifts Induced by Noise Exposure by RWM Administration

**FIGURE 3:**
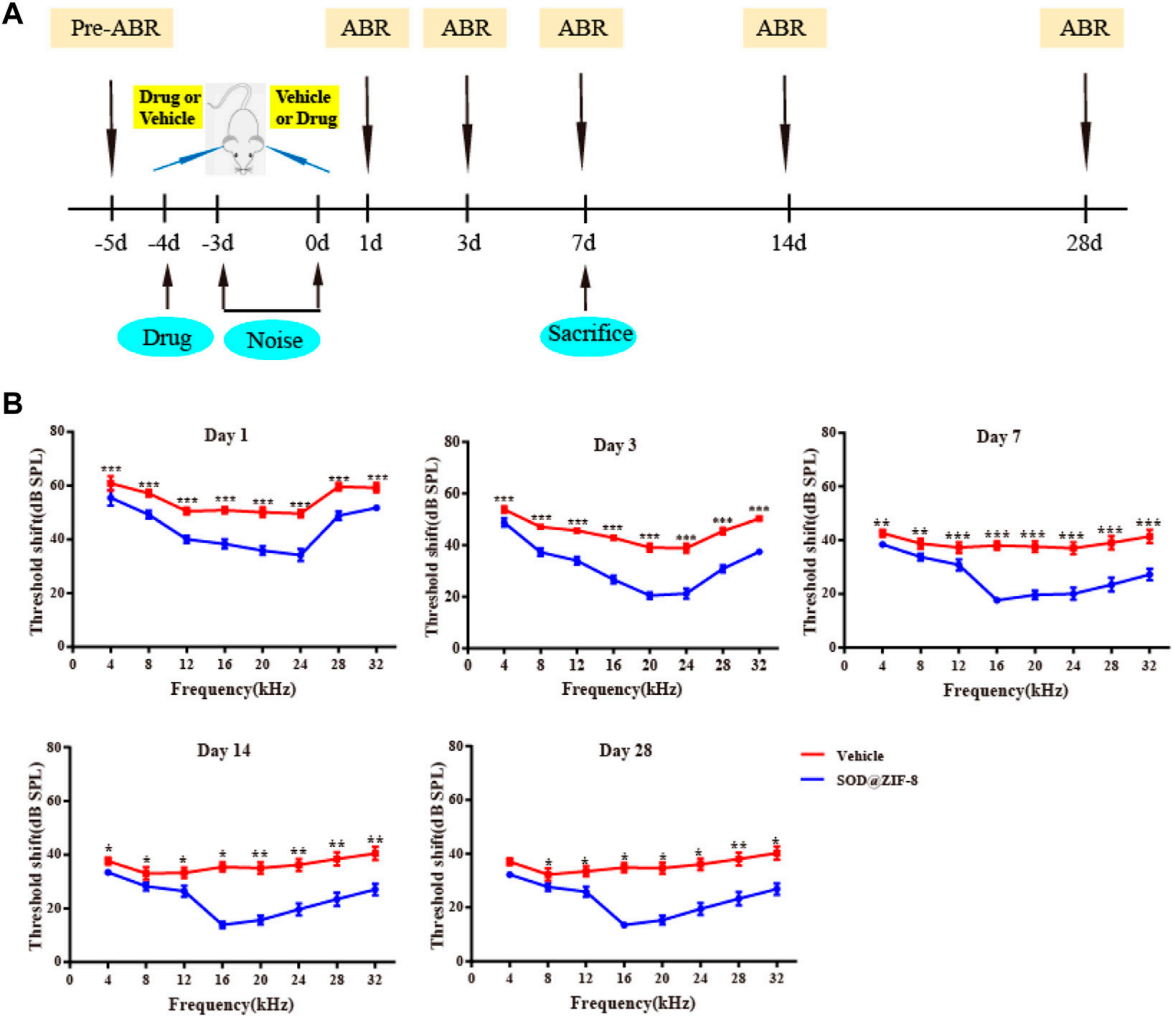
Superoxide dismutase (SOD)@zeolite imidazolate framework-8 (ZIF-8) protected against noise-induced hearing loss (NIHL). **(A)** Experimental design. **(B)** Graphs show mean threshold shifts (means ± standard error of mean) across all frequencies (4, 8, 12, 16, 20, 24, 28, and 32 kHz) analyzed at days 1, 3, 7, 14, and 28 in animals exposed to noise and treated with SOD@ZIF-8 or vehicle (*p < 0.05, **p < 0.01, and ***p < 0.001). Days 1, 3, and 7 (*n* = 24), days 14 and 28 (*n* = 6).

We have demonstrated that our noise stimulus (120–125 dB SPL white noise, 12 h/day for 3 consecutive days) can induce permanent threshold shifts (PTS) in Wistar rats (Supplementary Figure S1). The auditory thresholds were increased up to ∼80 dB at the frequency range of 4–32 kHz 1 day after noise exposure, with the most significant increase observed at 4 kHz. On day 7, thresholds dropped 10–20 dB, and they leveled off over during the upcoming 3 weeks. Subsequently, to explore the protection of SOD@ZIF-8 against NIHL, we performed ABR in rats before drug administration, and at 1, 3, 7, 14, and 28 days after noise exposure (Figure 3A). The baseline thresholds were ∼20 dB at all frequencies in all experimental animals and did not differ between bilateral ears (Supplementary Figure S2). As shown in Figure 3B, SOD@ZIF-8 significantly improved the hearing thresholds of all frequencies compared with the vehicle group, especially at mid-high frequencies. In the vehicle group, the mean threshold increased remarkably by approximately 50–60 dB at all frequencies on days 1, 3, and 7 after noise exposure. After the treatment of SOD@ZIF-8, the threshold shift was attenuated at all frequencies and time points. At days 1 and 3, there was a 20–55 dB threshold shift, whereas further reduction, which ranged from 20 to 30 dB, was observed on day 7 in the SOD@ZIF-8 group. Moreover, there were significant differences (*p* < 0.001) at frequencies in the range of 12–32 kHz at days 1, 3, and 7 between the vehicle and SOD@ZIF-8 groups. At days 14 and 28, the ABR threshold recovered slightly, but the difference between the two groups was still significant. This also suggests that the protective effect of the single dose of nanoparticles was long lasting.

The results of ABR suggested that SOD@ZIF-8 can attenuate auditory threshold shifts induced by noise exposure following RWM administration. Systemic administration is the main way to treat inner ear diseases, but the blood-labyrinth barrier (BLB) hampers the administration of an effective therapeutic dosage from reaching the inner ear. Particularly, some protein and nucleic acid drugs have low bioavailability owing to systemic administration. The method of RWM administration to the inner ear is a common local approach for drug delivery due to greater drug bioavailability (El Kechai et al., 2015). Studies have shown that the RWM is a semipermeable membrane with certain permeability and material transport function (Hellström et al., 1989; Goycoolea and Lundman, 1997). Microspheres (diameters in the range of 1 μm) can pass through the RWM of the rabbit (Goycoolea et al., 1988), and nanoparticles with diameters in the range of 170–190 nm can pass through the RWM of the rat (Martín- Saldaña et al., 2018). Additionally, literature indicated that noise exposure can induce alterations to the BLB, which can make it easier for drugs to reach the organ of Corti (Suzuki et al., 2002; Wu et al., 2014; Bielefeld and Kobel, 2019). Our results also demonstrated that SOD@ZIF-8 nanoparticles can cross the RWM and BLB following their administration. However, our study did not provide any direct evidence that the drug can pass through the RWM and BLB and the mechanism based on which it can pass through these anatomical structures.

### 3.3 SOD@ZIF-8 Decreased Hair Cell Loss Induced by Noise Exposure and Protected the Endocochlear Gap Junctions

**FIGURE 4:**
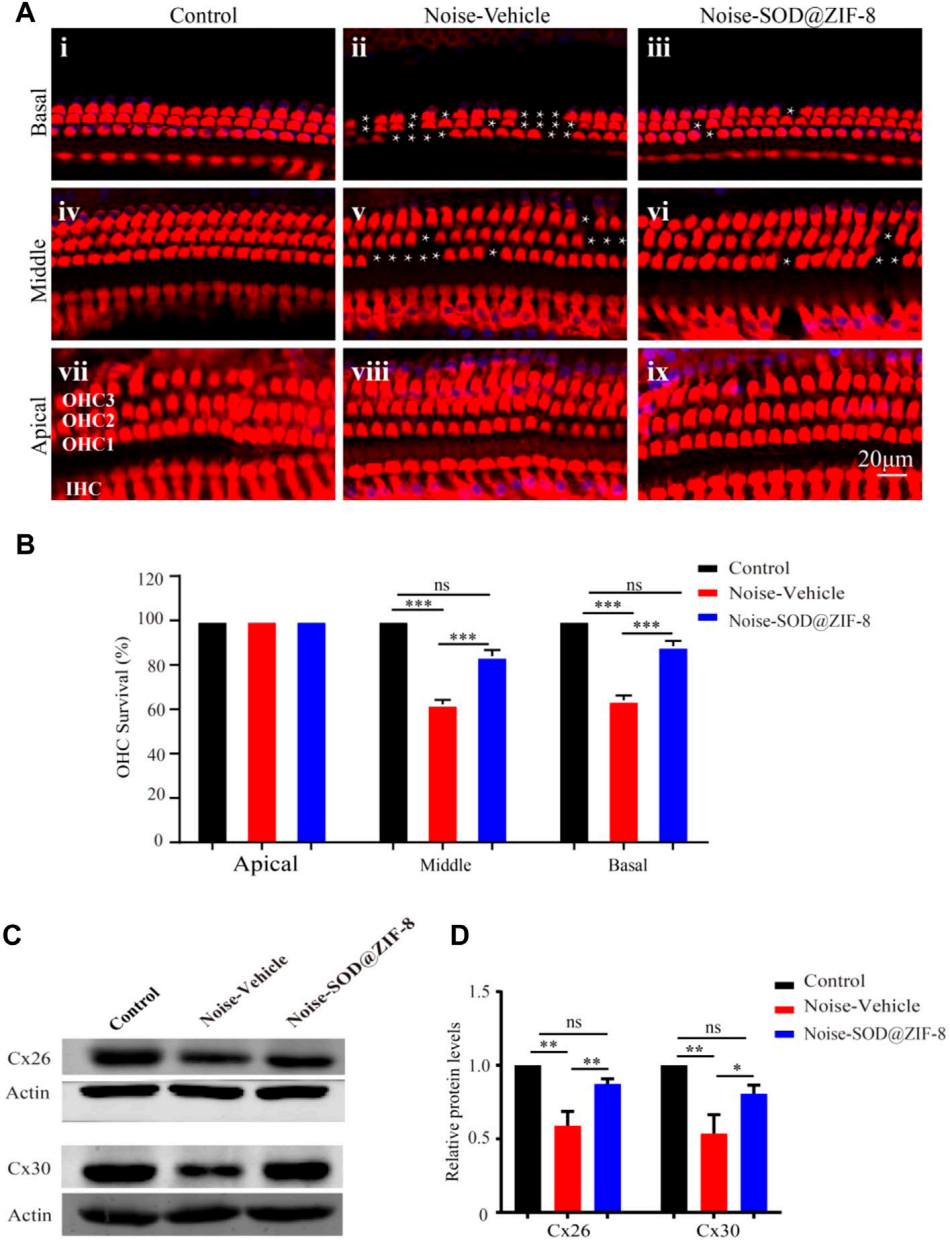
Superoxide dismutase (SOD)@zeolite imidazolate framework-8 (ZIF-8) protected cochlear hair cells (HCs) and endocochlear gap junctions from noise-induced hearing loss (NIHL). **(A)** Representative images of surface preparation of the organ of Corti in the basal–apical cochlear turn from three groups of rats (Control, Vehicle, SOD@ZIF-8). **(B)** Analysis of HC count in each group. **(C,D)** Protein expression levels and statistical analysis of Cx26 and Cx30 in the cochleae of various groups. The white asterisk represents the missing outer hair cells (OHCs). Cyan represents 4′6′-diamino-2-phenylindole, and red represents Myosin-VIIa. Data are presented as means ± standard error of mean (SEM) (*p < 0.05, **p < 0.01, and ***p < 0.001, ns: no statistical significance).

Outer hair cell (OHC) loss was the principal sign of PTS, which was induced by noise exposure (Neal et al., 2015). To explore the protective effect of SOD@ZIF-8 on the cochlear acoustic injury, we examined hair cell numbers in surface preparations of the basilar membrane on day 7 after noise exposure. We used Myosin-VIIa labeled cochlear sensory epithelia for hair cell counts. As shown in Figures 4Ai,iv,vii, HCs in each turn were arranged neatly, and there was no obvious deletion in the control group. In agreement with a previous study (Neal et al., 2015), noise exposure mainly caused OHC losses (marked with white asterisks in the middle and basal turns in Figures 4Aii,v). SOD@ZIF-8 distinctly reduced OHCs’ loss of the middle–basal turns (Figures 4Aiii,vi). OHC counts denoting hair cell survival in the middle and basal turn in the vehicle ears were ∼62 and ∼64%, respectively, compared with ∼83 and ∼88% in the same regions in the SOD@ZIF-8 ears, respectively (Figure 4B). There was no significant loss of OHC in the apical turns of all groups. Additionally, we only found the absence of individual inner HCs in the noise exposure group.

Furthermore, to confirm the protective effect of SOD@ZIF-8on cochlea, we conducted western blot analysis for endocochlear gap junctions Cx26 and Cx30. Cx26 and Cx30 are the main endocochlear gap junction subtypes, which allow intracellular signaling molecules to be released in the extracellular space (Stout et al., 2004). As shown in Figure 4D, there were significant decreases in the expressions of Cx26 and Cx30 in the noise–vehicle cochleae compared with the control group. However, cochleae with SOD@ZIF-8 administration showed enhancements in the expressions of Cx26 and Cx30. The results of statistical analysis also confirmed that the protective effect of SOD@ZIF-8 on the two gap junctions was statistically significant (Figure 4E). These demonstrated that noise exposure damaged cochlear gap junctions and that SOD@ZIF-8 had a protective effect on them. Altogether, our results showed that SOD@ZIF-8 could ameliorate the loss of OHCs and the reductions of Cx26 and Cx30 expressions induced by noise exposure.

### 3.4 SOD@ZIF-8 Reduced the Level of Cochlear ROS and Oxidative Damage

**FIGURE 5:**
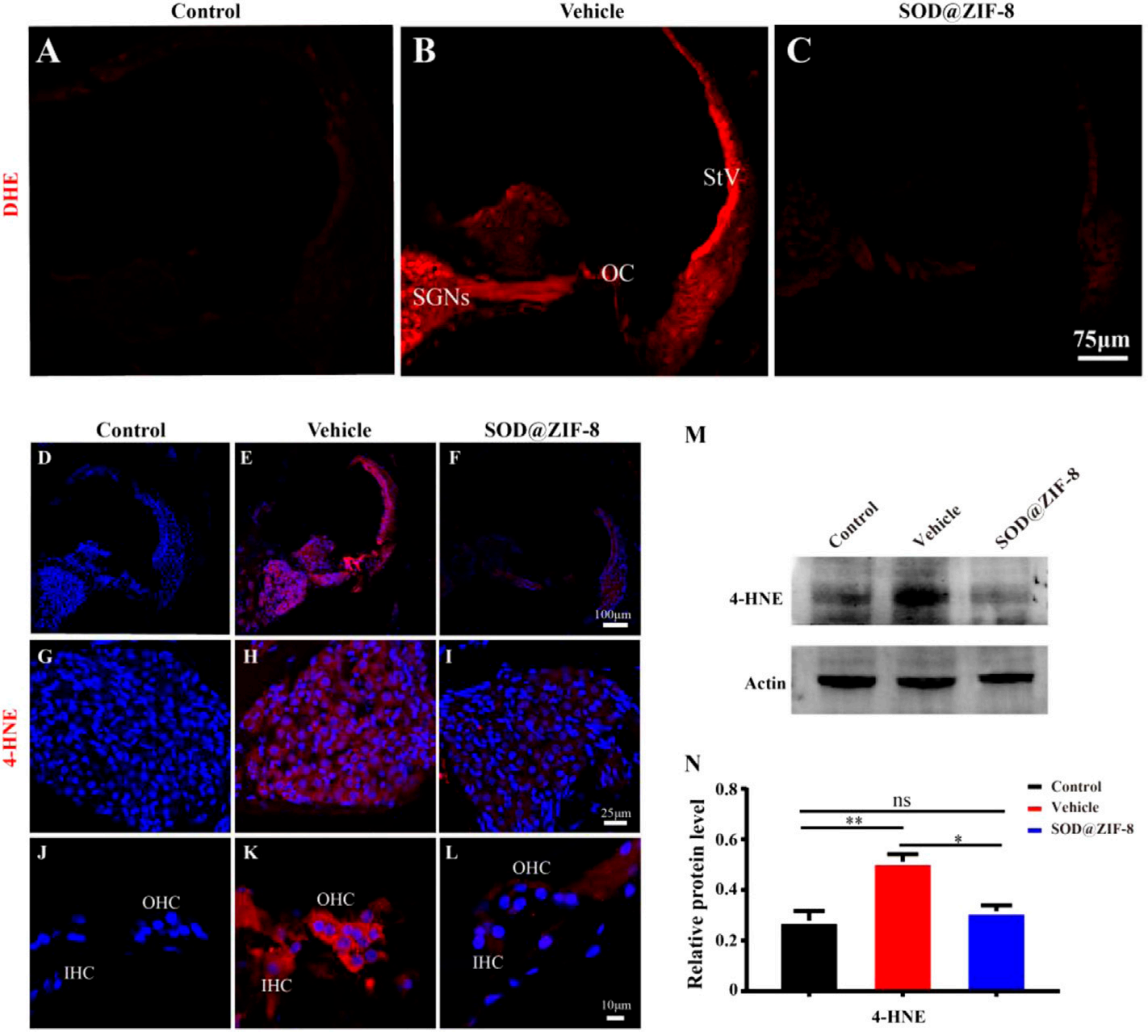
Superoxide dismutase (SOD)@zeolite imidazolate framework-8 (ZIF-8) administration reduced the levels of reactive oxygen species (ROS) and 4-hydroxynonenal (4-HNE) caused by noise. **(A–C)** Representative images of dihydroethidium staining in the middle–basal cochlear turns from three groups. **(D–F)** Representative images of 4-HNE staining in the middle–basal cochlear turns from three groups. 4-HNE fluorescence obviously changed in the spiral ganglion neurons (SGNs) (G–I) and organ of Corti (OC) **(J–L)**. **(M,N)** Protein expression level and statistical analysis of 4-HNE in the cochleae of various groups (StV: Stria Vascularis; OC: Organ of Corti; SGNs: Spiral Ganglion Neurons). Data (N) are presented as means ± standard deviation (SD) (*p < 0.05, **p < 0.01, ns: no statistical significance).

In physiological states, the production and clearance of ROS are in equilibrium. Excessive noise stimulation can cause the production of a large number of ROS and exceed the cochlear clearance ability, eventually leading to oxidative damage of cochlear tissues (Wu et al., 2020). After noise exposure, the cochlear tissue can produce ROS immediately, which will last for 7–10 days after noise exposure (Henderson et al., 1999). We conducted immunofluorescence on cochlear sections to detect O_2_^−^ (DHE staining) and the lipid peroxidation product 4-HNE to explore the protective effects of SOD@ZIF-8 on noise-induced oxidative stress (Fetoni et al., 2015). In the control group, DHE staining was faint (Figure 5A), whereas it dramatically increased in the vehicle group, especially in the spiral ganglion neurons (SGNs), the organ of Corti (OC), and the stria vascularis (StV) (Figure 5B). SOD@ZIF-8 administration remarkably decreased DHE fluorescence in the three abovementioned areas, owing to the effective ROS scavenging (Figure 5C). However, the staining of the group was still stronger than the control group (Figures 5A,C).

In parallel, in the vehicle group, acoustic trauma obviously increased 4-HNE immunolabeling compared with the control group, which indicated lipid peroxidative damage in the cochlea (Figures 5D,E). Figures 5H,K show that the fluorescence staining of 4-HNE was mainly concentrated in the SGNs, OC, and StV, that is, the same locations wherein the DHE staining was mainly concentrated. SOD@ZIF-8 significantly diminished 4-HNE expressions in the StV, SGNs and OC of the cochlea (Figures 5F,I,L). These results were consistent with the DHE staining outcomes. To confirm the immunofluorescence result, we performed immunoblotting analyses on 4-HNE. As shown in Figures 5M,N, the expression of 4-HNE increased in the vehicle cochleae and decreased markedly in the SOD@ZIF-8 cochleae. All of these results indicated that SOD@ZIF-8 possesses strong scavenging abilities for cochlear-redundant ROS and can efficiently alleviate cochlear oxidative damage caused by noise.

### 3.5 SOD@ZIF-8 Suppressed the Inflammation and Apoptosis in the Noise-Damaged Cochlae

**FIGURE 6:**
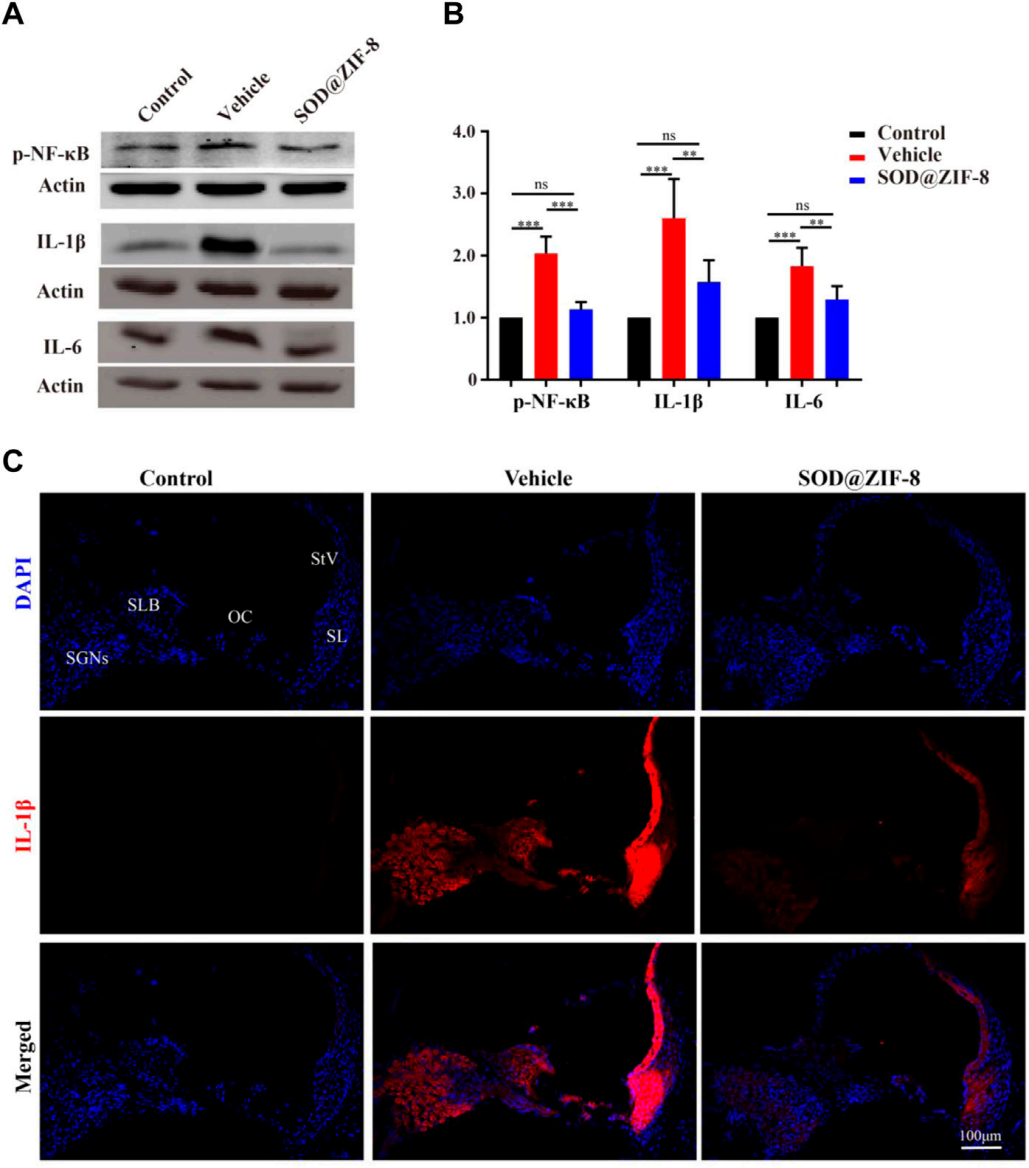
Superoxide dismutase (SOD)@zeolite imidazolate framework-8 (ZIF-8) administration counteracted inflammation in the cochlea caused by noise exposure. **(A,B)** Protein expression level and statistical analysis of phosphonuclear factor kappa B (p-NF-κB), interleukin (IL)-6, and IL-1β in the cochleae of various groups. **(C)** Representative images of IL-1 staining in the middle–basal cochlear turns from three groups (StV: Stria Vascularis; SL: Spiral Ligament; SLB: Spiral Limbus; OC: Organ of Corti; SGNs: Spiral Ganglion Neurons). Data are presented as means ± standard deviation (SD) (**p < 0.01, ***p < 0.001, ns: no statistical significance).

Studies have shown that nanoparticles that were injected to scavenge ROS have significant anti-inflammatory activity (Mu et al., 2019; Zhao et al., 2019). Recently, the role of pro-inflammatory factors in cochlear injury and its relationship with ROS have been addressed. ROS can cause the generation of proinflammatory cytokines, which can further lead to cochlear damage (Wakabayashi et al., 2010; Tan et al., 2016). Therefore, we examined the anti-inflammatory effects of SOD@ZIF-8 on NIHL on day 7. We detected the expression of p-NF-κB, a key regulator of inflammation, and pro-inflammatory cytokines IL-1β and IL-6 by western blotting. In our study, increased levels of p-NF-κB, IL-1β, and IL-6 were observed in noise-damaged cochlear tissues, and SOD@ZIF-8 treatment markedly inhibited these changes (Figures 6A,B **)**. As shown in Figure 6B, IL-1β was the most significantly altered; thus, we then further clarified the distribution of IL-1β in the cochlea via immunofluorescence. Compared with the control, increasing IL-1β immunolabeling was observed in SGNs, StV, spiral limbus (SLB), and in fibrocytes (types II and V) of the spiral ligament in cochlear from the noise-exposed rats (Figure 6C). SOD@ZIF-8 supplementation effectively blocked the noise-induced increase in IL-1β. All these results indicated obvious anti-inflammatory effects of SOD@ZIF-8 on noise-induced cochlear injury.

Additionally, to investigate whether SOD@ZIF-8 could relieve the apoptosis of the noise-damaged cochleae, we detected the proapoptotic protein bax and the antiapoptotic protein bcl2 by western blotting. After exposure to noise, the protein expression of bax was increased and the protein expression of bcl-2 was decreased, and SOD@ZIF-8 treatment restored these changes (Supplementary Figures S3A–D). This result indicated that SOD@ZIF-8 can restrain apoptosis caused by noise in the cochlea.

### 3.6 Probable Mechanism of SOD@ZIF-8 Mediated Nanotherapy for NIHL

**FIGURE 7:**
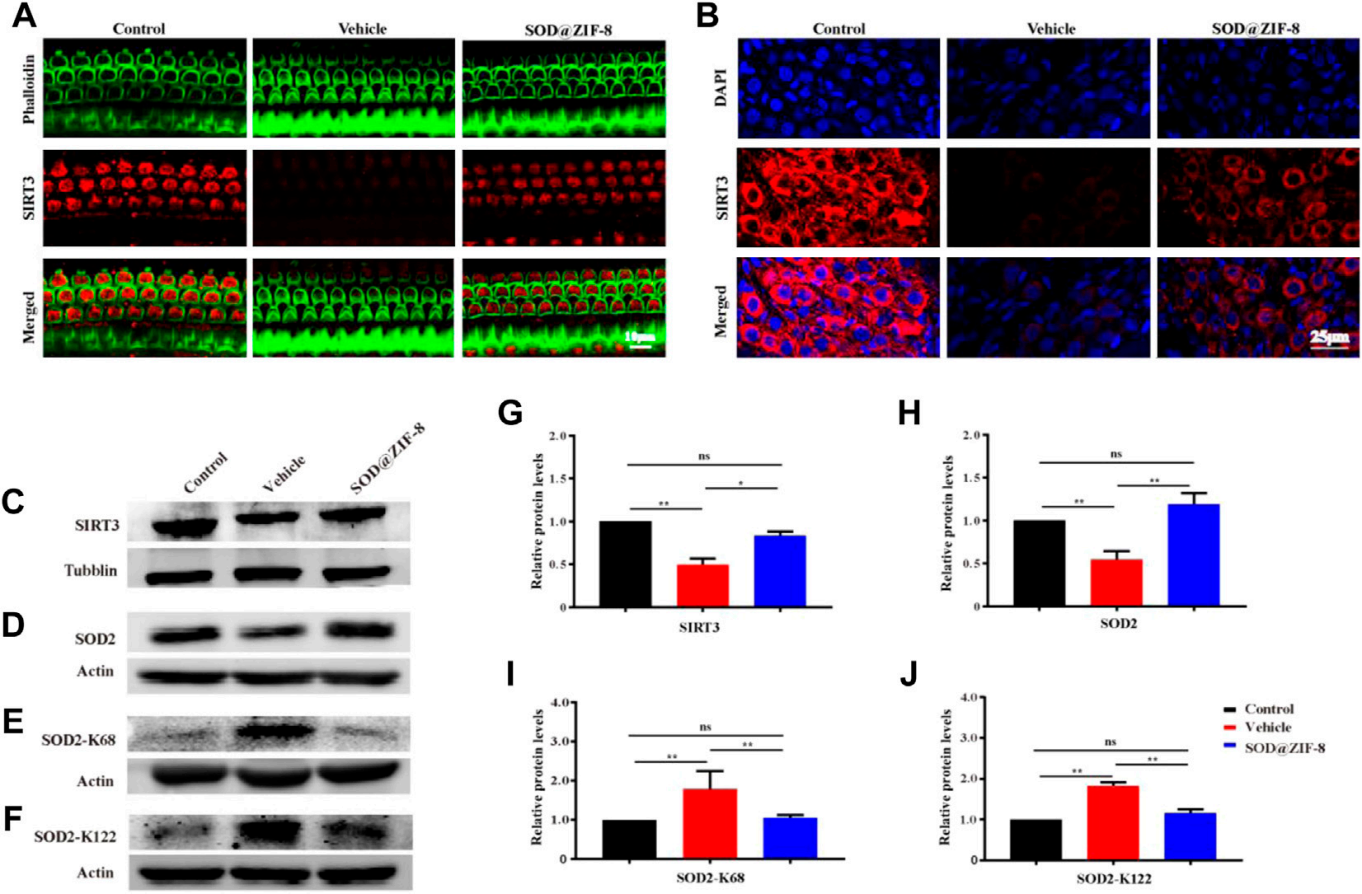
Superoxide dismutase (SOD)@zeolite imidazolate framework-8 (ZIF-8) administration counteracted the decrease of Sirtuin-3 (SIRT3) and SOD2 caused by noise. **(A)** Representative images of SIRT3 staining in spiral ganglion neurons (SGNs) of the middle–basal cochlear turns from three groups. **(B)** Representative images of SIRT3 staining in outer hair cells of the basal cochlear turns from three groups. **(C–J)** Protein expression levels and statistical analyses of SIRT3, SOD2, SOD2-K68, and SOD2-K122 in the cochleae of various groups. Data are presented as means ± standard deviation (SD) (*p < 0.05, **p < 0.01, ns: no statistical significance).

Furher, we explored the probable mechanism of SOD@ZIF-8 on protecting cochleae against noise-induced damage. It is well known that mitochondria are the main sources of ROS associated with ROS-induced oxidative damage (Raimundo et al., 2012), and our results suggested that SOD@ZIF-8 was effective in reducing ROS and oxidative damage in the noise-damaged cochlea. Sirtuin-3 (SIRT3), an oxidized nicotinamide adenine dinucleotide (NAD+)-dependent major mitochondrial deacetylase, was demonstrated to play a critical role in the regulation of ROS formation and in the control of inflammatory responses (Kurundkar et al., 2019). Additionally, studies have shown that the activation of SIRT3 has a protective effect on noise-damaged and aging cochleae in mice (Someya et al., 2010; Brown et al., 2014). To explore the effects of SOD@ZIF-8 on SIRT3, we detected the expression of SIRT3 via immunofluorescence and western blot analysis. Figure 7A shows representative images of SIRT3 staining in frozen slices of control, noise–vehicle, and noise-SOD@ZIF-8. In the control group, SIRT3 was strongly expressed in the cytoplasm of SGNs and it was significantly reduced after noise exposure. SOD@ZIF-8 administration remarkably increased the expression of SIRT3. Additionally, we conducted immunolabeling for SIRT3 with surface preparations and observed the same trend in OHCs of the middle–basal turns (Figure 7B). Furthermore, we verified immunofluorescence results by western blotting. Consistent with these results, SOD@ZIF-8 remarkably counteracted the noise-induced decreased protein levels of SIRT3 (Figures 7C,G).

Emerging evidence suggests that SOD2 is one of the most commonly known endogenous antioxidant enzymes and an important downstream protein of the SIRT3 signaling pathway. The activity of SOD2 is mediated by post-translational modification through lysine acetylation at K68 and K122 that inhibits its activity. More importantly, the mitochondrial deacetylase SIRT3 can deacetylate SOD2, which further enhances SOD2 activity. Therefore, we measured the protein expressions of SOD2, Ac-SOD2-K68, and acetylated Ac-SOD2-K122. The expression of SOD2 was significantly decreased in the cochleae on day 7 after noise exposure, and SOD@ZIF-8 administration restored the SOD2 protein levels (Figures 7D,H). Conversely, the levels of Ac-SOD2-K68 and Ac-SOD2-K122 after noise exposure were higher than control, while SOD@ZIF-8 treatment substantially diminished SOD2 acetylation (Figures 7E,F,I,J). Our results indicated that SOD@ZIF-8 could enhance the intracellular mitochondrial reduction system by increasing the protein levels of SIRT3 and SOD2 to improve the antioxidant capacity of the noise-exposed cochlea.

### 3.7 Biosafety Assessment of SOD@ZIF-8

The biosafety analysis of cochlea local application SOD@ZIF-8 was further investigated 10 days after administration. We studied the histomorphology of the main organs in the experimental rat group by H&E staining to determine the biosafety of SOD@ZIF-8. 10 days after administration, we did not find any obvious pathological changes in the sections of the heart, brain, liver, spleen, and kidney (Supplementary Figure S4). Furthermore, there was no significant difference in peripheral blood biochemical parameters, including ALT, AST, BUN, CR, and CK, between the groups (Table 1). These results suggest that SOD@ZIF-8 produced no significant side effects on the structures or functions of main organs. This also indicated that the biosafety of local application of SOD@ZIF-8 is reliable.

**TABLE 1:** Serum alanine aminotransferase (ALT), aspartate aminotransferase (AST), blood urea nitrogen (BUN), creatinine (Cr), and creatine kinase (CK) activities at day 28. Data are expressed as means ± standard deviation (SD).

	ALT (U/L)	AST (U/L)	BUN (mg/dl)	Cr (umol/L)	CK (U/L)
Control	86.01 ± 3.71	128.10 ± 5.37	20.00 ± 2.74	33.33 ± 4.33	1241.00 ± 65.58
Noise	86.67 ± 2.73	129.90 ± 6.12	20.73 ± 3.73	32.64 ± 4.74	1222.00 ± 86.07

## 4 Conclusion

In summary, we have demonstrated that the treatment of SOD@ZIF-8 significantly alleviated the levels of ROS, oxidative damage, and inflammation in the cochlea after acoustic trauma. Moreover, our study indicates that SOD@ZIF-8 administration efficiently protected the cochlea from hearing loss caused by noise exposure, at least partially, by modulating the SIRT3/SOD2 signaling pathway. Therefore, SOD@ZIF-8 may be a novel therapeutic option for NIHL. The presented approach constitutes an entirely new approach and can be used to treat other ROS-related inner ear diseases.

## Data Availability

The original contributions presented in the study are included in the article/Supplementary Material, further inquiries can be directed to the corresponding authors.
