# Supplementary material for: Superoxide dismutase@zeolite Imidazolate Framework-8 Attenuates Noise-Induced Hearing Loss in Rats
Source: Front Pharmacol. 2022 May 16;13:885113. doi: 10.3389/fphar.2022.885113 (PMC9159373; doi:10.3389/fphar.2022.885113)
Supplement: Supplementary file 1 [file DataSheet1.PDF]

Supporting Information

Superoxide dismutase@zeolite imidazolate framework-8 attenuates noise-induced hearing loss in rats

Yan Zhang[a,c, #], Qing Li[b, #], Chengzhou Han[a, 2], Fang Geng[a, 2], Sen Zhang[b], Yan Qu[*, a], Wenxue Tang[*, b]

[a] Department of Otolaryngology, The Third Hospital of Hebei Medical University, Shijiazhuang, Hebei 050051, China.

[b] Center for Precision Medicine of Zhengzhou University, Zhengzhou 450052, China.

c Department of Otolaryngology, Tangshan People' s Hospital, Tangshan, Hebei 063000, China.

#These authors contributed equally.

*Corresponding author.

E-mail: 1473760147@qq.com (Yan Qu); twx@zzu.edu.cn (Wenxue, Tang).

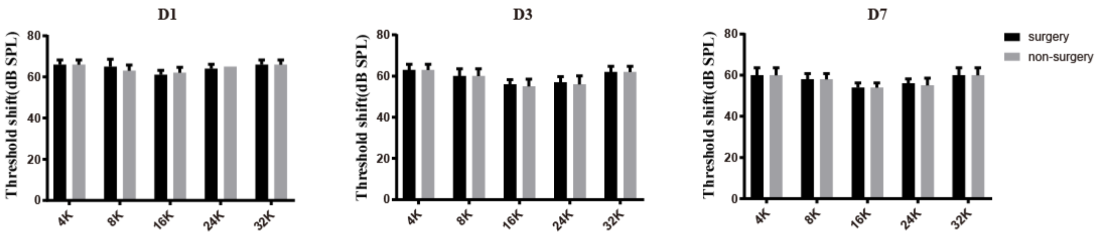

Fig. S1 ABR threshold shift in bilateral ears (surgery and non-surgery) at different time points after noise exposure.

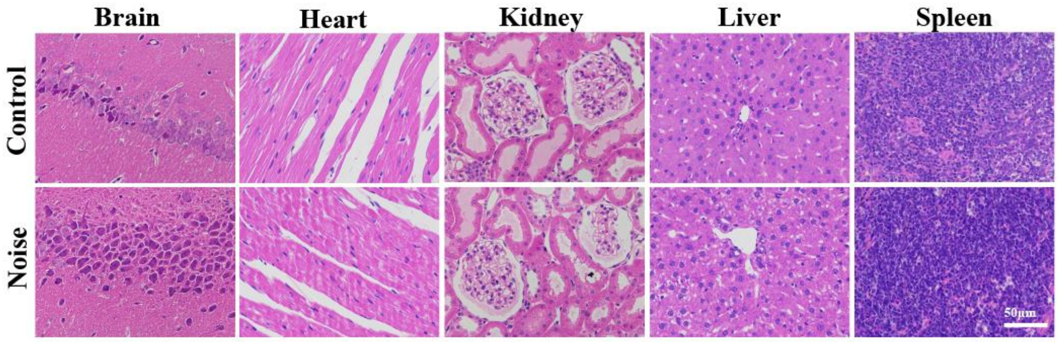

Fig. S2 Representative images of H&E stained brain, heart, kidney, liver and spleen sections at day 28.

	ALT (U/L)	AST (U/L)	BUN (mg/dl)	Cr (umol/L)	CK (U/L)
Control	86.01±3.71	128.10±5.37	20.00±2.74	33.33±4.33	1241.00±65.58
Noise	86.67±2.73	129.90±6.12	20.73±3.73	32.64±4.74	1222.00±86.07

Table 1 Serum alanine aminotransferase (ALT), aspartate aminotransferase (AST), blood urea nitrogen (BUN), creatinine (Cr) and creatine kinase (CK) activities at day 28. Data are expressed as means ± standard deviation (SD).
